# LC-YOLOmatch: a novel scene segmentation approach based on YOLO for laparoscopic cholecystectomy

**DOI:** 10.3389/frai.2025.1706021

**Published:** 2025-12-08

**Authors:** Hong Long, Yuancheng Shao, Mini Han Wang, Fengshi Jing, Yuqiao Chen, Shuai Xiao, Jia Gu

**Affiliations:** 1 Department of Gastrointestinal Surgery, The First Affiliated Hospital, Hengyang Medical School, University of South China, Hengyang, China; 2 Faculty of Data Science, City University of Macau, Taipa, Macau SAR, China; 3 Zhuhai Precision Medical Center, Zhuhai People's Hospital, The Affiliated Hospital of Beijing Institute of Technology, Zhuhai Clinical Medical College of Jinan University, Zhuhai, China; 4 School of Medicine, The University of North Carolina at Chapel Hill, Chapel Hill, NC, United States; 5 Suzhou Ultimage Health Technology Co., Ltd., Suzhou, China

**Keywords:** laparoscopic sensing, surgical AI, real-time detection, multi-scale feature fusion, semi-supervised learning, image segmentation

## Abstract

**Introduction:**

Laparoscopy is a visual biosensor that can obtain real-time images of the body cavity, assisting in minimally invasive surgery. Laparoscopic cholecystectomy is one of the most frequently performed endoscopic surgeries and the most fundamental modular surgery. However, many iatrogenic complications still occur each year, mainly due to the anatomical recognition errors of surgeons. Therefore, the development of artificial intelligence (AI)-assisted recognition is of great significance.

**Methods:**

This study proposes a method based on the lightweight YOLOv11n model. By introducing the efficient multi-scale feature extraction module, DWR, the real-time performance of the model is enhanced. Additionally, the bidirectional feature pyramid network (BiFPN) is incorporated to strengthen the capability of multi-scale feature fusion. Finally, we developed the LC-YOLOmatch semi-supervised learning framework, which effectively addresses the issue of scarce labeled data in the medical field.

**Results:**

Experimental results on the publicly available Cholec80 dataset show that this method achieves 70% mAP50 and 40.8% mAP50-95, reaching a new technical level and reducing the reliance on manual annotations.

**Discussion:**

These improvements not only highlight its potential in automated surgeries but also significantly enhance assistance in laparoscopic procedures while effectively reducing the incidence of complications.

## 1 Introduction

Cholecystectomy is one of the most common surgeries in general surgery, with approximately 1.2 million such operations performed globally each year. Among them, about 92% are carried out through laparoscopic techniques (Jones et al., 2025), marking the wide application of minimally invasive surgical technology in modern medicine. However, due to differences in economic development levels, medical equipment conditions, and surgical training systems among various regions, the incidence of surgical complications varies significantly (Okoroh and Riviello, 2021). For young surgeons, mastering this routine surgery is not only a basic requirement for career development but also an important guarantee for patient safety.

To help young doctors master the operation skills more quickly and reduce the risk of surgical complications, many research teams have been dedicated to developing real-time surgical recognition systems in recent years (Cao et al., 2023; Tang et al., 2024). These systems can analyze and guide the key steps of the surgical process in real time through artificial intelligence technology, thereby shortening the learning curve and improving surgical efficiency (Kou et al., 2023). Currently, various deep learning-based methods have been applied in this field. For instance, segmentation systems based on the U-Net architecture can accurately identify anatomical structures in the surgical field (Kihira et al., 2022); while transformer-based models, with their strong ability to extract global features, have demonstrated excellent performance in complex scenarios (Tang et al., 2023). Additionally, many studies have applied mature detection models such as YOLOv5, YOLOv8, and Segment Anything Model (SAM) to surgical video analysis, achieving very satisfactory results (Ping et al., 2023) (Sivakumar et al., 2025). Currently, the new generation of YOLO11 series has shown outstanding speed and accuracy in various detection and segmentation tasks (Ali and Zhang, 2024). This project adopts the YOLO11n model and makes improvements to meet the requirements of real-time accuracy for laparoscopic surgery. We enhance the efficiency of multi-scale feature extraction by combining the DWR module with the core module C3K2 of YOLO, and introduce BiFPN to improve the ability of multi-scale feature fusion, achieving a more efficient and accurate model.

Although this method has made significant technical progress, its practical application still faces many challenges. The primary issue is the excessively high cost of data annotation (Vijayanarasimhan and Grauman, 2009). Although there are abundant sources of surgical videos, the fine annotation of multiple organs within them requires a considerable amount of time and effort from professional surgeons. A single high-quality annotated image may take several hours or even longer. Therefore, even for a relatively small dataset (such as one containing 1,000 images), it demands a huge investment of human and material resources. Moreover, since surgical video data often contain sensitive patient privacy information, publicly available datasets are extremely limited. Even when some datasets are accessible, their annotation standards vary significantly among different research teams, making it difficult to achieve generalization (Wei et al., 2018). This scarcity of data and inconsistency in annotation severely restrict the development and promotion of related technologies.

To address the aforementioned issues, we have developed a framework based on semi-supervised learning. Specifically, we first train an initial weight for our improved model using a small amount of high-quality labeled data. Then, we use this model to predict the unlabeled data and generate pseudo-labels. Meanwhile, we balance the quality and quantity of the pseudo-labels through confidence screening. Subsequently, we apply strong augmentation to the unlabeled images, predict them using the model weights, and supervise them with the pseudo-labels. We optimize the model through one-norm regularization, effectively reducing the reliance on manual labeling. Experimental results show that this method not only significantly reduces the labeling cost but also greatly enhances the utilization of large-scale unlabeled data by the model.

In conclusion, the deep learning method proposed in this study demonstrates high accuracy and efficient data utilization capabilities, and has good clinical promotion value. It is expected to become an important tool for assisting surgeons' training and surgical operations in the future.

The key contributions of this work include the following:

- This study employed all the videos from the Cholec80 dataset to construct a comprehensive dataset by uniformly sampling images across different surgical stages and diverse scenarios.
- We have developed an innovative method called LC-YOLO, which has improved the accuracy and real-time performance of scene segmentation and target detection in laparoscopic cholecystectomy.
- We innovatively constructed the LC-YOLOmatch framework, reducing the high reliance on manually labeled data and enhancing the utilization rate of labeled data.
- It clearly expounds the motivation for seeking an automated-assisted laparoscopic cholecystectomy solution, providing a method for artificial intelligence-assisted surgery.

## 2 Related work

This section reviews the relevant literature on artificial intelligence in laparoscopic cholecystectomy, laying the theoretical foundation for the LC-YOLOmatch framework proposed in this study. This framework aims to enhance the segmentation accuracy of target images in laparoscopic cholecystectomy and effectively address the issue of insufficient manual annotation in existing studies.

### 2.1 CNN-based methods

In recent years, convolutional neural network (CNN) has been widely used for the automatic segmentation and recognition of anatomical structures in laparoscopic videos (Jalal et al., 2023; Kitaguchi et al., 2020; Ward et al., 2021). Shinozuka et al. (2022) adopted EfficientNet-B7 as the basic algorithm to develop a deep convolutional neural network (CNN) model, which can accurately identify the surgical stages during laparoscopic cholecystectomy. Lee et al. (2024) combined CNN with long short-term memory networks (LSTM) on this basis, conducting time series analysis on video frame sequences to determine the surgical progress in real time. Madani et al. (2022) designed two CNN models: GoNoGoNet for identifying safe and dangerous areas, and CholeNet for recognizing anatomical structures. The model performance was evaluated through 10-fold cross-validation, achieving good intersection over union (IoU) and F1 scores. However, all these methods rely on high-quality manual annotations, and the real-time performance in complex scenarios still needs to be optimized.

### 2.2 YOLO-based methods

The YOLO model has been widely used in laparoscopic videos for real-time detection and localization of key anatomical structures (Pan et al., 2024; Lai et al., 2023). Compared with traditional CNN models, YOLO has a significant advantage in speed and is suitable for real-time intraoperative applications. Tokuyasu et al. (2021) developed a model based on YOLOv3 to identify four key anatomical landmarks (common bile duct, cystic duct, lower edge of the medial segment of the left liver, and Rouviere's sulcus) during cholecystectomy. Although the average precision in quantitative evaluation was not high, expert surgeons subjectively assessed that the model could successfully identify key anatomical landmarks in most test videos. Yang et al. (2024) introduced a channel attention (CA) mechanism into the backbone network of YOLOv7, which improved mAP, precision, and recall. Smithmaitrie et al. (2024) used YOLOv7 to detect two anatomical landmarks, Rouviere's sulcus and the lower edge of liver segment IV, and deployed it in the operating room for real-time detection and visualization guidance of anatomy. Generally, YOLO is mostly used for object detection, and its image segmentation capability is often overlooked.

### 2.3 Pseudo-labeling technology

In medical image segmentation, pseudo-labels are particularly suitable for scenarios where data annotation is costly and there is an abundance of unlabeled data, such as laparoscopic surgery images (Wu et al., 2023; Wang et al., 2021). Due to the characteristics of laparoscopic cholecystectomy images, including high noise, complex anatomical structures, and low contrast, pseudo-labels can effectively expand the training data and enhance the model's generalization ability in complex scenarios. Owen et al. (2022) introduced a computer vision model that was trained to identify key structures in laparoscopic cholecystectomy images, namely the cystic duct and cystic artery. This model utilized label relaxation to address the ambiguity and variability in annotations and adopted pseudo-label self-supervised learning to leverage unlabeled data for training. The model was trained using 3,050 labeled and 3,682 unlabeled frames of cholecystectomy images and achieved an IoU of 65% and a target presence detection F1 score of 75%. Three expert surgeons verified the model's output and found it to be accurate and promising.

## 3 Materials and methods

### 3.1 Dataset

**Figure 1:**
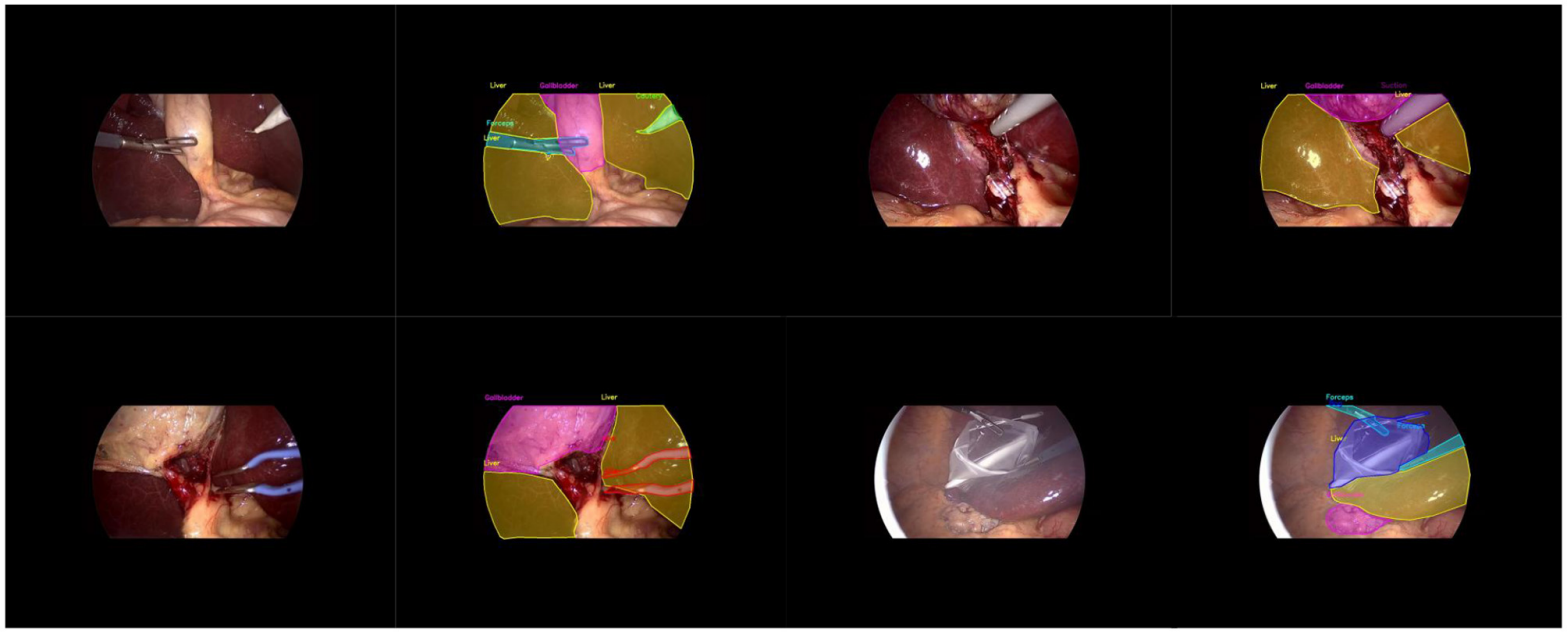
Diagram illustrating the display of images and labels.

Our main dataset is derived from the renowned Cholec80, which originated from the research of A.P. (Twinanda et al., 2016). It contains 80 laparoscopic cholecystectomy surgery videos, along with organized and labeled information at different stages of the surgeries. To achieve better results, we adopted the labeling method from the research of Tashtoush et al. (2025). We manually extracted 544 images from the first three videos and conducted detailed polygonal labeling on them. For the remaining 77 videos, to avoid repetitive labeling of similar images, we automatically extracted one frame every minute from each surgery video, and removed interfering images that were not within the surgical field, such as severe lens contamination, lens cleaning, and the lens moving out of the surgical area. In total, we obtained 2,550 unlabeled images. We allocated 323 labeled images from the first segment of the Cholec80 video to the training dataset, 133 standard images from the second segment to the validation dataset, and 88 standard images from the third segment to the test dataset. As shown in Figure 1, it is the display of our image and manual annotation.

### 3.2 LC-YOLO architecture

**Figure 2:**
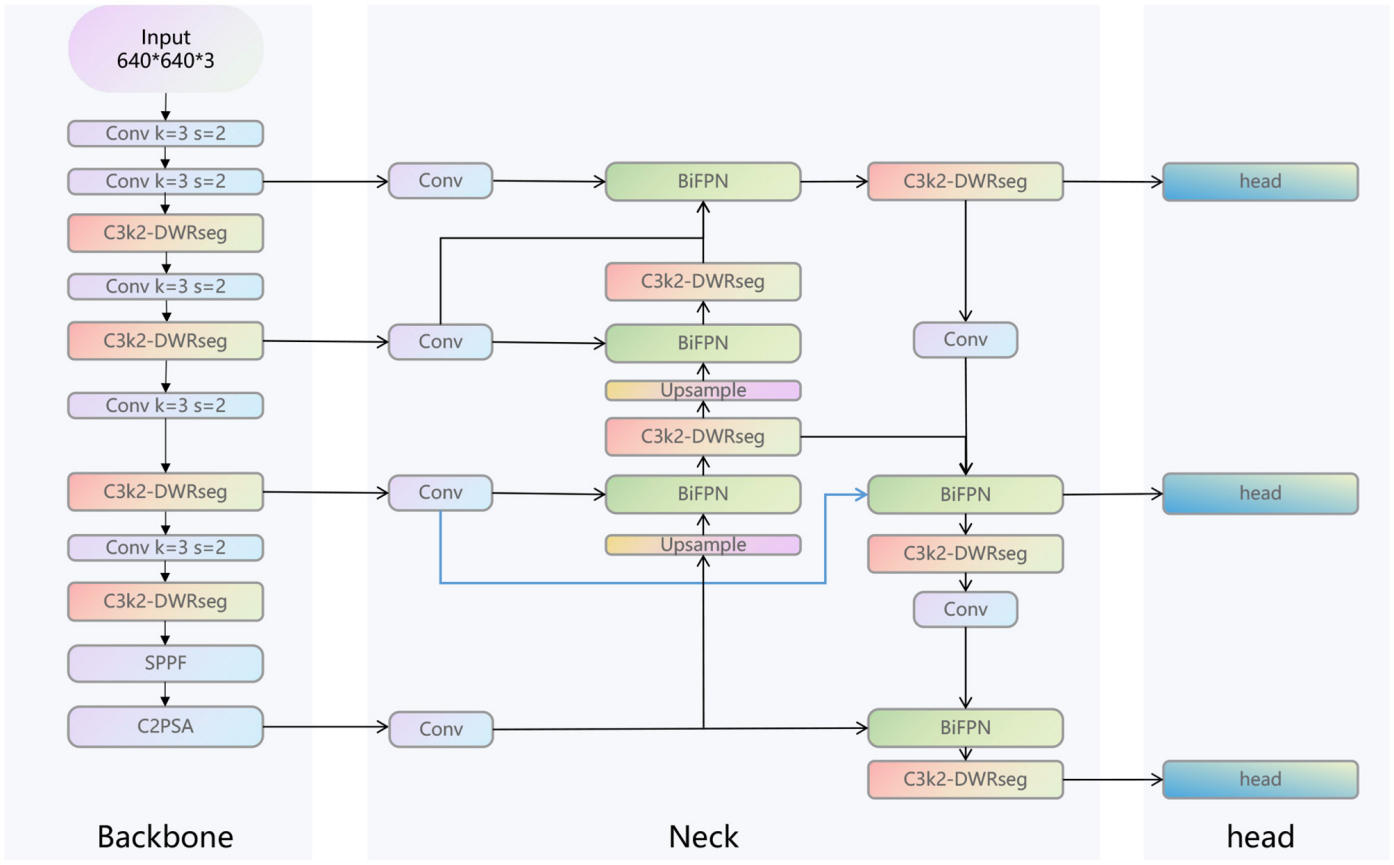
The structure of LC-YOLO.

As shown in Figure 2, the overall structure diagram of our LC-YOLO model is presented. This model is based on the yolov11n model. Before extracting the features of the 4th, 6th, and 10th layers of the backbone network in the neck network, we add a convolutional layer to uniformly adjust the number of channels to 256. This is to enable better input of multi-scale features into the BIFPN layer for more complex feature fusion. To enhance the model's ability to detect small targets and perform precise positioning, we add a set of modules for extracting features from the lower layers of the backbone network, such as the edges, textures, corner points and other basic features of the image. We replace all the C3K2 modules in the model with C3K2-DWRseg modules to enhance the model's multi-scale feature extraction ability and efficiency to adapt to the real-time requirements of medical applications. We replace concat with BiFPN to improve the ability of multi-scale feature fusion and achieve more precise model performance.

### 3.3 C3K2-DWRseg module

**Figure 3:**
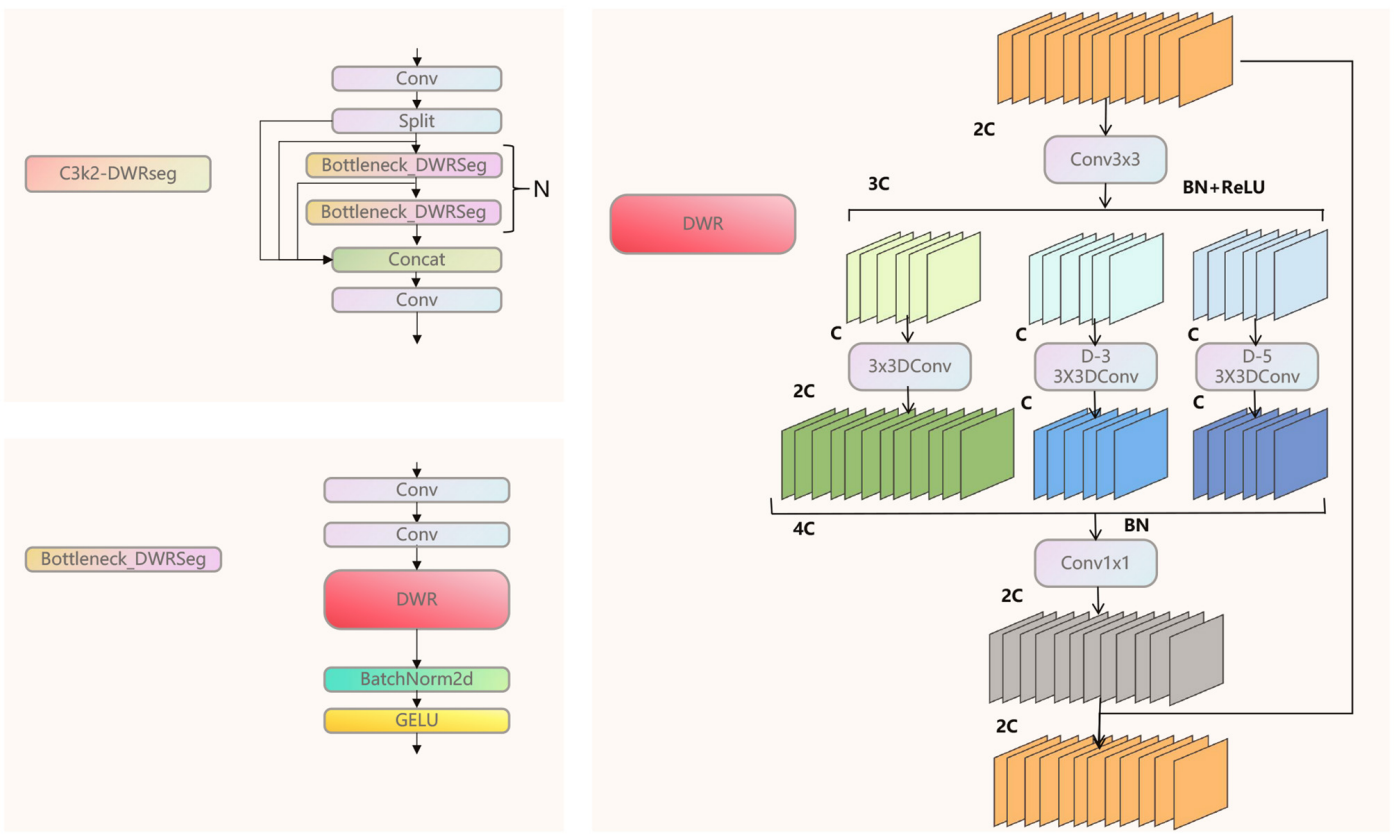
The structure of C3K2-DWRseg module.

As shown in Figure 3, we analyzed the core structure of the C3K2-DWRseg module. DWR is an innovative expansion-style residual proposed by Wei et al. (2018). This module consists of three branches, each of which uses different dilated convolutions to expand the receptive field. The dilated rates are 1, 3, and 5. The traditional Bottleneck is used to control the dimension of features, aiming to reduce the computational cost while retaining important features. We innovatively combined the Bottleneck with the DWR module to form the Bottleneck-DWRSeg. This approach reduces the computational cost while rapidly extracting multi-scale features, highlighting the real-time performance of the model. Then, we replaced the Bottleneck in C3K2 with our Bottleneck-DWRSeg, ultimately forming the C3K2-DWRseg module.

### 3.4 BiFPN module

**Figure 4:**
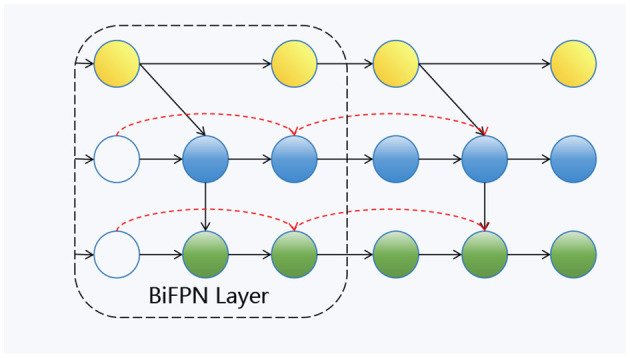
The structure of BiFPN module.

As shown in Figure 4, this is our Bi-FPN layer (Tan et al., 2020). In contrast to the traditional feature pyramid network (FPN), which has a unidirectional flow, multi-scale feature fusion seeks to combine features of varying resolutions more efficiently. Unlike FPN, which is restricted to a single input node, Bi-FPN nodes can process information from multiple inputs, facilitating the integration of both low-level and high-level semantic features. Within the Bi-FPN structure, feature fusion occurs in both bottom-up and top-down directions, allowing the model to capture cross-scale features more effectively. In our proposed model, multi-layer features are utilized as inputs for each Bi-FPN layer.

### 3.5 LC-YOLOmatch framework

**Figure 5:**
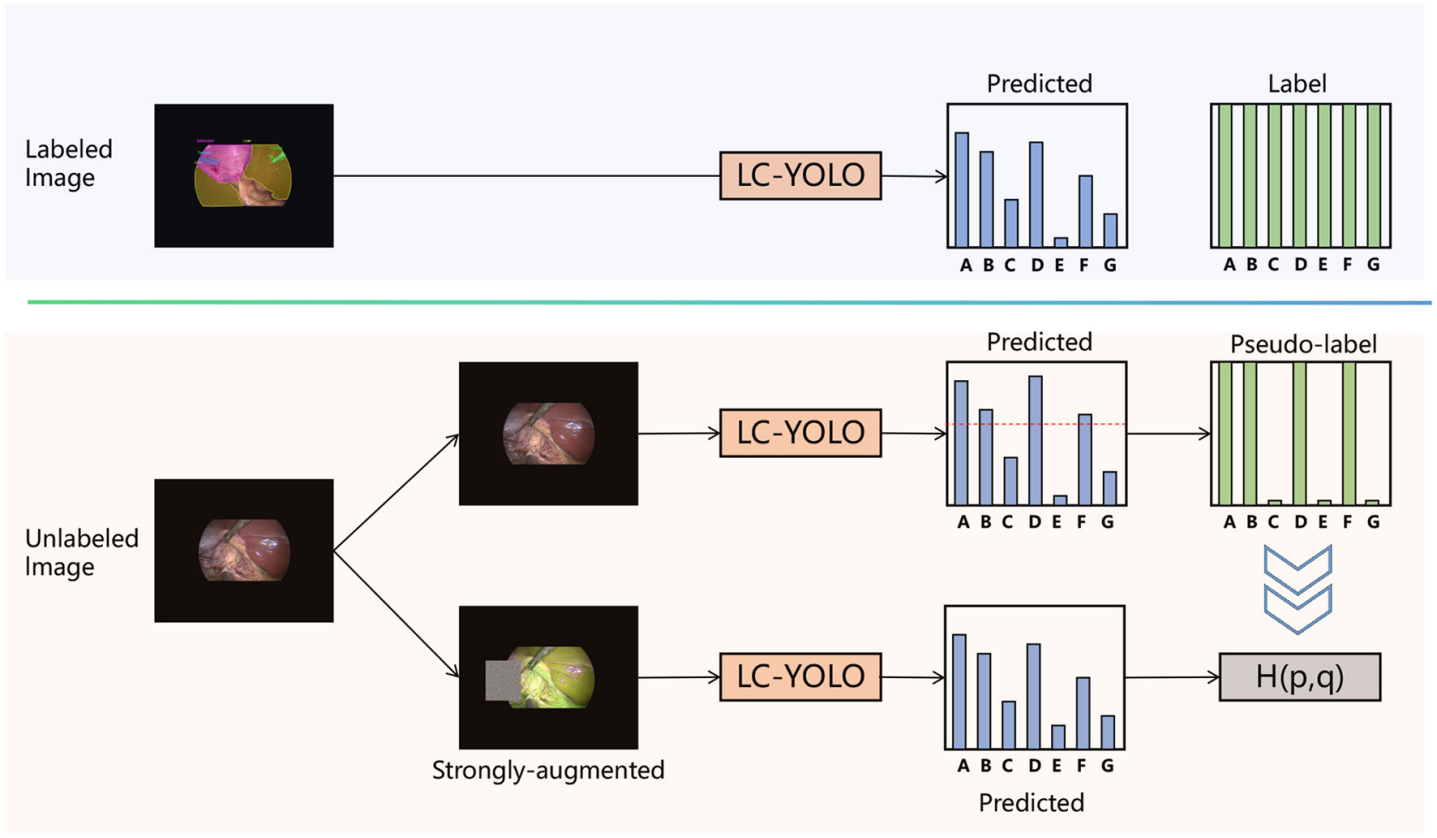
The framework of LC-YOLOmatch.

To address the issue of scarce manually labeled images, we aim to make rational use of unlabeled images. Drawing on the framework of FixMatch (Sohn et al., 2020), as shown in Figure 5, we have designed the LC-YOLOmatch framework. Firstly, we train the initial weights using the labeled images in LC-YOLO. Then, we use this weight model to predict the unlabeled images. To solve the problem of the accuracy of pseudo-labels, we utilize the confidence function provided by YOLO to filter out the qualified pseudo-labels. Using the qualified pseudo-labels as supervision, we again use the initial weight model to predict the strongly enhanced processed unlabeled images, achieving consistent regularization and further enhancing the model's capabilities.

## 4 Experiment

### 4.1 Experimental evaluation

For assessing the model's performance, we utilize the standard quantification metrics commonly employed in YOLO. In this context, TP denotes the count of true positive samples, FP refers to the number of false positive samples, and FN indicates the number of false negative samples. Precision (P) evaluates the fraction of model-predicted positive samples that are genuinely true positives. A higher value signifies more accurate predictions by the model and fewer instances of negative samples being misclassified as positive. The Equation 1 is presented as follows:


$$\begin{array}{l} P=\frac{TP}{TP+FP} \end{array}$$ (1)

R indicates the ratio of all actual positive samples that the model is capable of accurately predicting as positive. A higher value reflects greater coverage of positive samples by the model and fewer overlooked positive instances. The Equation 2 is formulated as follows:


$$\begin{array}{l} R=\frac{TP}{TP+FN} \end{array}$$ (2)

mAP50 represents the mean average precision when the intersection over union (IoU, the ratio of intersection to union) exceeds 0.5. It is used to evaluate whether the overlap between the predicted mask and the actual mask bounding box exceeds 50%. This metric can comprehensively assess the model's performance in segmentation tasks.

mAP50-95 denotes the average of the mean average precision computed across varying IoU thresholds (ranging from 0.5 to 0.95 with increments of 0.05). This measure offers a more extensive evaluation of the model's performance under diverse detection rigor levels, demands greater overall capabilities from the model, and exhibits enhanced robustness and generalization power.

In order to balance the trade-off between precision and recall, the F1 value is defined in the Equation 3 as:


$$\begin{array}{l} F1=2\times \frac{\text{P}\times \text{R}}{\text{P}+\text{R}} \end{array}$$ (3)

### 4.2 Experimental setup

The proposed model was implemented on a computer equipped with a 12GB memory RTX 4080 graphics card using Pytorch 1.21.1 and Python 3.9.7. The optimizer used SGD, the maximum number of training epochs was set to 200, the learning rate was set to the default value of 0.01, and the image input was 640*640.

Our LC-YOLO function architecture is developed based on the Ultralytics code library (Jocher et al., 2022). For the weak enhancement, the default data augmentation settings of YOLOv11 are adopted. For the strong enhancement, color jittering is used: randomly adjust the brightness, contrast, saturation and hue of the image, with the parameters being a brightness factor of 0.5, a contrast factor of 0.5, a saturation factor of 0.5 and a hue factor of 0.2. The larger the value of these factors, the more obvious the color change of the image; random grayscale conversion: The default probability is set to 0.2 to convert the image to a grayscale image; Gaussian blur: The default probability is set to 0.5 to perform Gaussian blur processing on the image; occlusion: Randomly select a rectangular area on the image and mask to occlude it, and the pixel values of this area are randomly filled, while the values of the corresponding area in the mask are set to 255.

Finally, the loss function of our semi-supervised framework LC-YOLOmatch consists of two parts. One part is the supervised loss ℓlc-yolo, and the other part is the semi-supervised loss ℓlcyolo-match. The total loss function of the model is ℓ_lc-yolo_+βℓ_lcyolo-match_, where β is a fixed scalar hyperparameter used to balance the weights of the two parts of the loss.

For labeled segmentation samples, ℓ_ℓ*c*-*yolo*_ also employs the cross-entropy loss function to calculate the difference between the predicted results of the labeled images after weak enhancement and the true labels. *N* is the batch size of the labeled samples; *H* represents the cross-entropy; *p*_*n*_ is the true label distribution of the sample *z*_*n*_, which is a pixel-level label distribution; *p*_ℓ*c*-*yolo*_(*y*|α(*z*_*n*_)) is the model's prediction distribution for the weakly enhanced labeled image α(*z*_*n*_), which is also a pixel-level prediction distribution.

The Equation 4 is defined as:


$$\begin{array}{l} l_{\text{lc-yolo}}=\frac{1}{N}\overset{N}{\underset{n=1}{\sum }}H\left(p_{n},p_{\text{lc-yolo}}\left(y|\alpha \left(z_{n}\right)\right)\right) \end{array}$$ (4)

For unlabeled segmented samples, the ℓ_lc-yolo-match_ algorithm first predicts the pseudo labels for the unlabeled images after weak enhancement. Then, it predicts the same images after strong enhancement and calculates the cross-entropy loss between the prediction results and the pseudo labels. To ensure the reliability of the pseudo labels, only when the maximum prediction probability of the model for the weakly enhanced images is greater than the preset threshold τ, will the pseudo label be retained and the loss be calculated. Here, μ*N* is the batch size of the unlabeled samples, μ is the ratio of unlabeled images to labeled images; *q*_*b*_ is the predicted distribution of the model for the weakly enhanced unlabeled images α(*u*_*b*_), which is a pixel-level prediction distribution; ${\hat{q}}_{b}$ is the pseudo label, that is, ${\hat{q}}_{b}=\text{argmax}\left(q_{b}\right)$, where the position with the maximum value for each pixel is taken as the pseudo label; *A*(*u*_*b*_) represents the image after strong enhancement of the unlabeled image *u*_*b*_. The Equation 5 is defined as:


$$\begin{array}{l} l_{\text{lc-yolomatch}}=\frac{1}{\mu N}\overset{\mu N}{\underset{b=1}{\sum }}1\left(\max\left(q_{b}\right)\geq \tau \right)H\left({\hat{q}}_{b},p_{\text{lc-yolomatch}}\left(y|A\left(u_{b}\right)\right)\right) \end{array}$$ (5)

Through this modified loss function, the LC-YOLOmatch method can effectively utilize both labeled and unlabeled data to enhance the performance of the image segmentation model.

## 5 Results

### 5.1 Comparative experiment

Firstly, to evaluate the performance of our enhanced model, we conducted both comparative and ablation studies. To ensure the fairness and consistency of the experiments, during the experimental phase, we adopted a uniform experimental setup and dataset across all trials. To minimize potential bias caused by parameter differences, all participating YOLO models utilized the smallest model variant. We employed the Wilcoxon signed-rank test to conduct pairwise comparisons of all image measurement indicators to evaluate the performance differences between the model and the optimized model. Meanwhile, we excluded categories with only two samples to ensure the reliability of the statistical analysis. The results showed that all the indicators included in the analysis presented *P* < 0.05, indicating significant statistical significance. The YOLO models were initially designed for detection tasks. Starting from YOLOv5 7.0 (Jocher et al., 2022), a segmentation function was introduced. The segmentation models selected for comparison included YOLOv5n, YOLOv6n, YOLOv8n, YOLOv9t, YOLOv10n, and YOLOv11n. The training set used 323 images manually annotated by us. We adopted the method of data augmentation to increase the sample size of the training set and ensured the consistency of the training set images. We simulated the frequent interference such as camera shake, smoke, and blood stains that occur during laparoscopic surgery operations. We used random rotation by 90 degrees and image blurring as the image enhancement mode to simulate the surgical scene. We generated two variants for each image. After preprocessing, we obtained a training set comprising 948 images, which was approximately three times the size of the original dataset.

**Figure 6:**
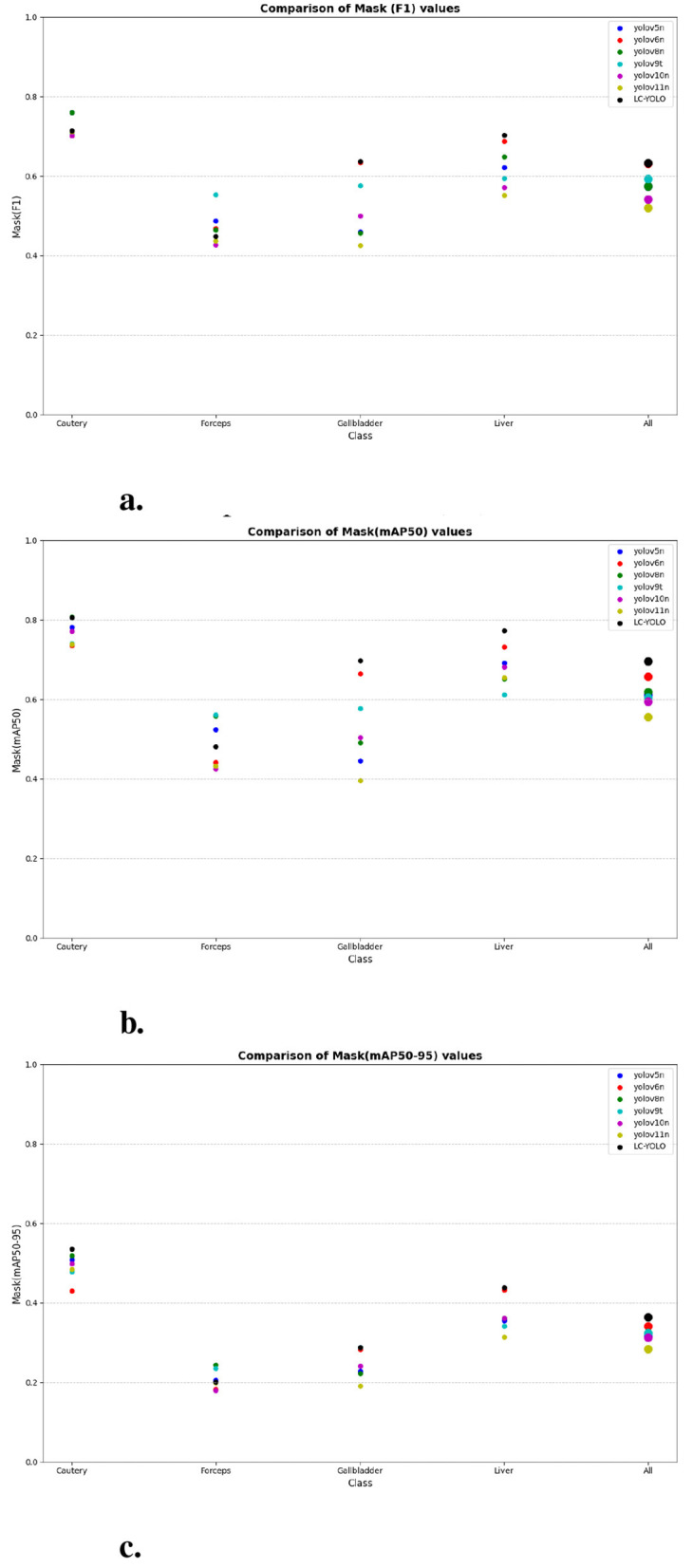
The scatter plot of the comparative experiment. **(a)** Comparison of mask (F1) values. **(b)** Comparison of mask (mAP50) values. **(c)** Comparison of mask (mAP50-95) values.

As shown in Tables 1–7, we obtained the results of each comparison model through five operations while maintaining the same division of the training set, validation set, and test set. Among them, the number of allis and bag instances in the test set is too small, so we do not make a separate comparison. The value of the All classification in YOLO is simply the average of the values of each classification, without considering the impact of different instance numbers of each classification, resulting in a deviation in the results. We recalculated and adopted the average based on the instance numbers. As shown in Figures 6a–c, the results are the comparison graphs of F1 value, mAP50, and mAP50-95 for each model. It can be seen that our LC-YOLO segmented 280 instances in 88 images, and it achieved the highest values in all categories. The F1 value was 21.7% higher than that of the base model YOLOv11n and 0.3% higher than that of the second-place yolov6n. The mAP50 was 25.2% higher than that of YOLOv11n and 5.9% higher than that of yolov6. The mAP50-95 was 28.1% higher than that of YOLOv11n and 6.7% higher than that of yolov6. The improvement in performance is very significant. At the same time, we observed that after multiple iterations of YOLO, its performance in the image segmentation project did not improve with the version iterations, indicating a huge room for optimization.

**Table 1:** The result of YOLOv5n.

**YOLOv5n**	**Images**	**Instances**	**Mask (P)**	**Mask (R)**	**Mask (F1)**	**Mask (mAP50)**	**Mask (mAP50-95)**
All	88	280	0.519	0.636	0.574	0.611	0.315
Allis	2	2	0.000	0.000	0.000	0.014	0.009
Bag	2	2	0.832	0.500	0.622	0.500	0.060
Cautery	43	44	0.799	0.724	0.760	0.782	0.509
Forceps	43	59	0.418	0.593	0.487	0.524	0.206
Gallbladder	65	65	0.409	0.523	0.460	0.445	0.229
Liver	78	108	0.531	0.769	0.621	0.691	0.355

**Table 2:** The result of YOLOv6n.

**YOLOv6n**	**Images**	**Instances**	**Mask (P)**	**Mask (R)**	**Mask (F1)**	**Mask (mAP50)**	**Mask (mAP50–95)**
All	88	280	0.637	0.639	0.631	0.657	0.341
Allis	2	2	0.088	0.500	0.150	0.100	0.034
Bag	2	2	0.824	0.500	0.635	0.496	0.198
Cautery	43	44	0.866	0.585	0.702	0.736	0.430
Forceps	43	59	0.545	0.407	0.469	0.441	0.184
Gallbladder	65	65	0.584	0.692	0.634	0.665	0.283
Liver	78	108	0.633	0.750	0.687	0.731	0.433

**Table 3:** The result of YOLOv8n.

**YOLOv8n**	**Images**	**Instances**	**Mask (P)**	**Mask (R)**	**Mask (F1)**	**Mask (mAP50)**	**Mask (mAP50–95)**
All	88	280	0.675	0.518	0.575	0.619	0.324
Allis	2	2	1.000	0.000	0.000	0.131	0.004
Bag	2	2	1.000	0.000	0.000	0.499	0.010
Cautery	43	44	0.856	0.682	0.760	0.808	0.518
Forceps	43	59	0.655	0.373	0.465	0.559	0.244
Gallbladder	65	65	0.579	0.385	0.457	0.492	0.222
Liver	78	108	0.668	0.63	0.649	0.651	0.361

**Table 4:** The result of YOLOv9t.

**YOLOv9t**	**Images**	**Instances**	**Mask (P)**	**Mask (R)**	**Mask (F1)**	**Mask (mAP50)**	**Mask (mAP50–95)**
All	88	280	0.535	0.674	0.593	0.604	0.324
Allis	2	2	0.000	0.000	0.000	0.000	0.000
Bag	2	2	0.492	0.492	0.492	0.249	0.0499
Cautery	43	44	0.718	0.695	0.706	0.740	0.479
Forceps	43	59	0.493	0.627	0.553	0.561	0.236
Gallbladder	65	65	0.527	0.631	0.576	0.577	0.288
Liver	78	108	0.499	0.731	0.594	0.612	0.342

**Table 5:** The result of YOLOv10n.

**YOLOv10n**	**Images**	**Instances**	**Mask (P)**	**Mask (R)**	**Mask (F1)**	**Mask (mAP50)**	**Mask (mAP50–95)**
All	88	280	0.437	0.730	0.541	0.594	0.313
Allis	2	2	0.105	0.500	0.176	0.0865	0.026
Bag	2	2	0.426	0.500	0.462	0.252	0.101
Cautery	43	44	0.605	0.841	0.702	0.771	0.499
Forceps	43	59	0.340	0.576	0.427	0.425	0.180
Gallbladder	65	65	0.414	0.646	0.500	0.504	0.241
Liver	78	108	0.444	0.806	0.571	0.681	0.362

**Table 6:** The result of YOLOv11n.

**YOLOv11n**	**Images**	**Instances**	**Mask (P)**	**Mask (R)**	**Mask (F1)**	**Mask (mAP50)**	**Mask (mAP50–95)**
All	88	280	0.431	0.668	0.520	0.556	0.284
Allis	2	2	0.000	0.000	0.000	0.000	0.000
Bag	2	2	0.772	0.5	0.622	0.502	0.1
Cautery	43	44	0.658	0.773	0.711	0.739	0.484
Forceps	43	59	0.34	0.627	0.437	0.433	0.2
Gallbladder	65	65	0.355	0.55	0.426	0.395	0.191
Liver	78	108	0.436	0.731	0.551	0.655	0.314

**Table 7:** The result of LC-YOLO.

**LC-YOLO**	**Images**	**Instances**	**Mask (P)**	**Mask (R)**	**Mask (F1)**	**Mask (mAP50)**	**Mask (mAP50–95)**
All	88	280	0.586	0.712	0.633	0.696	0.364
Allis	2	2	0.217	0.500	0.303	0.224	0.091
Bag	2	2	0.545	0.500	0.521	0.498	0.150
Cautery	43	44	0.714	0.727	0.714	0.806	0.536
Forceps	43	59	0.497	0.407	0.448	0.482	0.201
Gallbladder	65	65	0.529	0.800	0.637	0.697	0.289
Liver	78	108	0.623	0.824	0.703	0.773	0.438

Our LC-YOLO model still performed outstandingly in the subcategories. It ranked first in the cautery, gallbladder, and liver categories. Specifically, in the cautery category, our *mPA*50 was 3.1% higher than that of the second-place yolov5n, and our *mPA*50-95 was 3.5% higher than that of yolov8n. In the gallbladder category, our *F*1 was 0.47% higher than that of the second-place yolov6n, and our *mPA*50 was 4.8% higher than that of the second-place model. Our *mPA*50-95 was 0.35% higher than that of yolov8. In the liver category, our *F*1 was 2.3% higher than that of yolov6n, and our *mPA*50 was 5.7% higher than that of yolov6n. Our *mPA*50-95 was 1.2% higher than that of yolov6n. Although we did not achieve the top ranking in the forceps category, we still improved the *F*1 by 2.5%, *mAP*50 by 11.3%, and *mAP*50-95 by 0.5% compared to the original model YOLOv11n. These figures indicate that our LC-YOLO model has more precise and stable performance in the segmentation of laparoscopic cholecystectomy scenes, which can better assist surgeries and has certain clinical significance.

### 5.2 Ablation study

We conducted ablation experiments based on the YOLOv11n as the baseline model to systematically evaluate the impact of each module architecture on the model performance. The same training set, validation set, and test set division method as mentioned above was adopted in the experiments. As shown in Table 8, we summarized the results based on 88 images and 280 instance segmentation. The results show that our complete model LC-YOLO performs the best in overall performance, ranking first in recall rate, F1 value, mAP50, and mAP50-95 metrics. In the ablation experiments, the introduction of the BiFPN module or the DWRseg module alone significantly improved the performance compared to the baseline model YOLOv11n. Specifically, in the sub-classification tasks, YOLO+BiFPN performed best in the Cautery classification; YOLO+DWRseg performed best in the Forceps classification; while LC-YOLO, which integrates both the BiFPN and DWRseg modules, excelled in the Gallbladder and Liver classifications, especially achieving the best results in the crucial Gallbladder and Liver anatomical organ classification tasks in gallbladder removal surgery.

**Table 8:** This is the result of the ablation experiment.

**Class**	**Model**	**Images**	**Instances**	**Mask (P)**	**Mask (R)**	**Mask (F1)**	**mAP50**	**mAP50–95**
All	YOLOv11n	88	280	0.431	0.668	0.520	0.556	0.284
	YOLO + BiFPN	88	280	**0.701**	0.581	0.626	0.649	0.359
	YOLO + DWRseg	88	280	0.559	0.700	0.614	0.665	0.364
	LC-YOLO	88	280	0.586	**0.712**	**0.633**	**0.696**	**0.364**
Cautery	YOLOv11n	43	44	0.658	**0.773**	0.711	0.739	0.484
	YOLO + BiFPN	43	44	**0.840**	0.716	**0.774**	**0.814**	0.523
	YOLO + DWRseg	43	44	0.820	0.726	0.770	0.770	0.497
	LC-YOLO	43	44	0.714	0.727	0.714	0.806	**0.536**
Forceps	YOLOv11n	43	59	0.340	**0.627**	0.437	0.433	0.200
	YOLO + BiFPN	43	59	**0.671**	0.322	0.434	0.478	0.189
	YOLO + DWRseg	43	59	0.480	0.542	**0.509**	**0.485**	**0.220**
	LC-YOLO	43	59	0.497	0.407	0.448	0.482	0.201
Gallbladder	YOLOv11n	65	65	0.355	0.550	0.426	0.395	0.191
	YOLO + BiFPN	65	65	**0.651**	0.574	0.610	0.644	**0.358**
	YOLO + DWRseg	65	65	0.460	0.723	0.562	0.643	0.315
	LC-YOLO	65	65	0.529	**0.800**	**0.637**	**0.697**	0.289
Liver	YOLOv11n	78	108	0.436	0.731	0.551	0.655	0.314
	YOLO + BiFPN	78	108	**0.685**	0.667	0.687	0.681	0.381
	YOLO + DWRseg	78	108	0.565	0.787	0.653	0.750	0.422
	LC-YOLO	78	108	0.623	**0.824**	**0.703**	**0.773**	**0.438**

The optimal value is displayed in bold.

### 5.3 Comparison experiment with pseudo-labels

**Figure 7:**
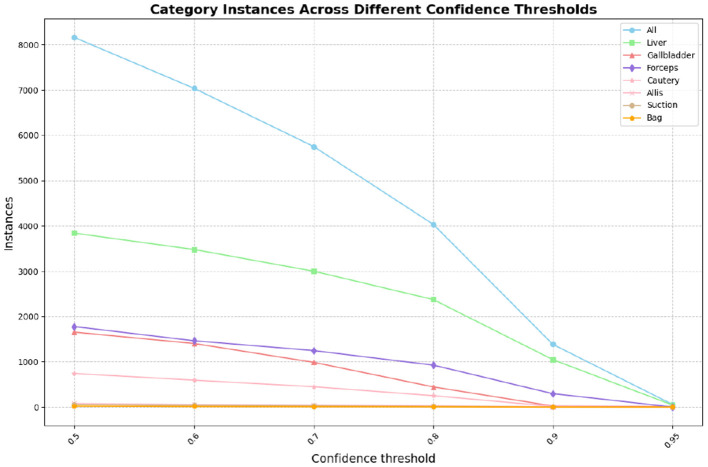
The number of pseudo-labels generated by different confidence levels.

**Figure 8:**
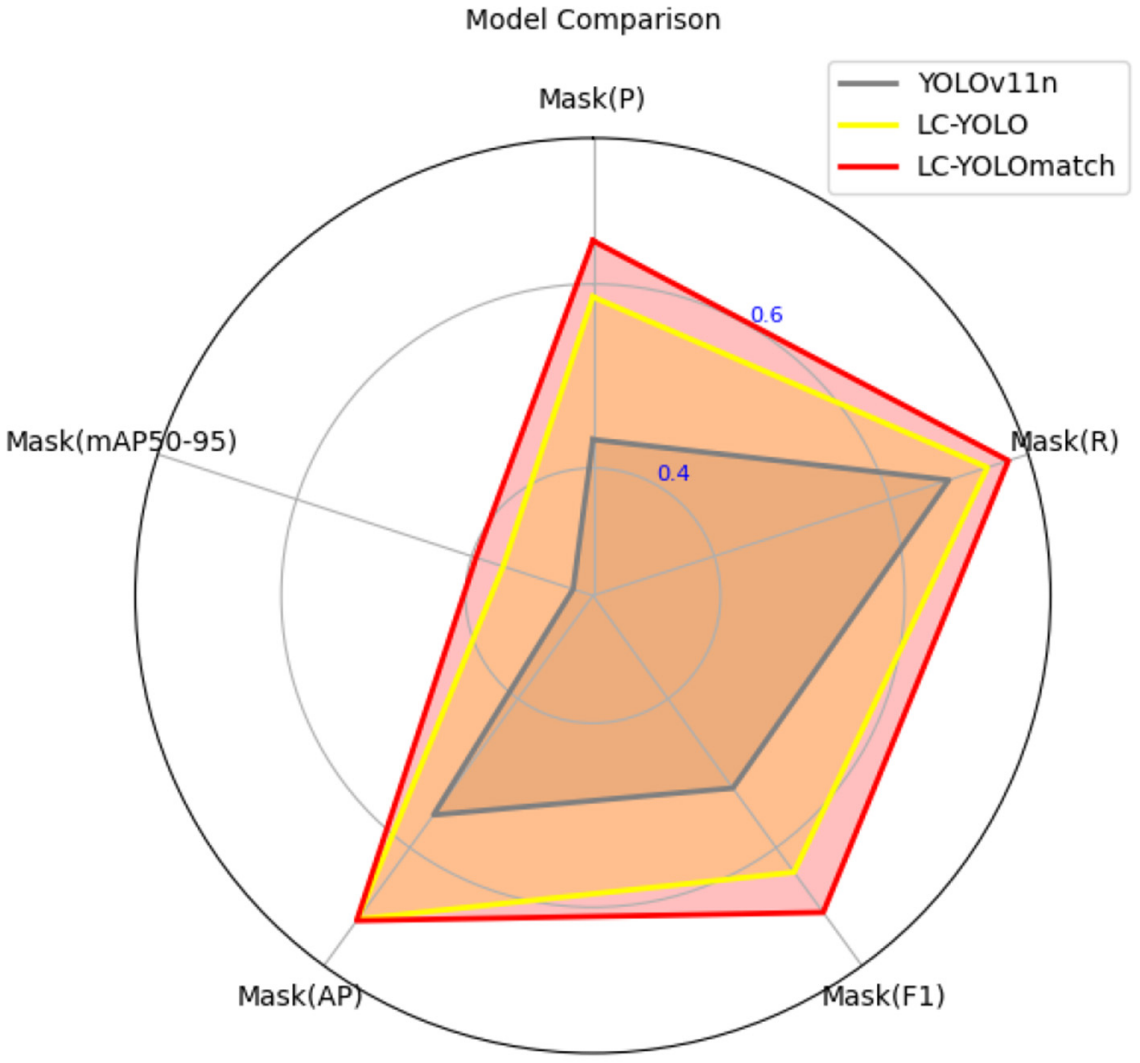
The comparison chart of model performance.

**Figure 9:**
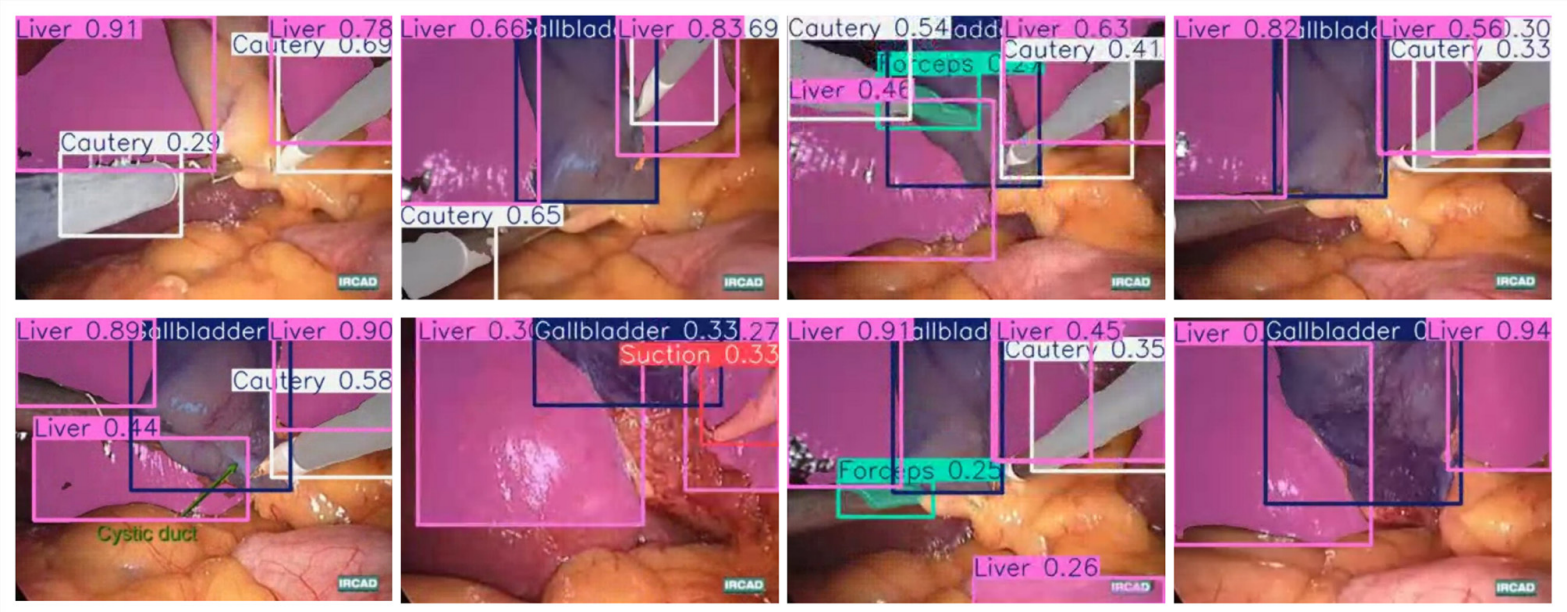
The standard laparoscopic cholecystectomy video test sample diagram.

In this part, we introduce LC-YOLO into our LC-YOLOmatch framework and use unlabeled images to further optimize the model. We generate pseudo-labels for the unlabeled images by setting different confidence thresholds τ, as shown in Figure 7, which presents the number of pseudo-label instances generated from 2,550 unlabeled images within the confidence interval of 0.5 to 0.95. Although a higher confidence threshold can filter out more accurate labels, it also leads to a significant loss of annotations, resulting in a linear decrease in the number of annotations. We set the confidence threshold τ to 0.6, with 323 manually labeled images in the training set, and the number of unlabeled images is set to 0 times, two times, three times, and eight times the labeled data, respectively. The validation set and test set are divided as described above. As shown in Table 9, our LC-YOLOmatch framework can effectively utilize unlabeled data resources to improve the model. Based on the overall instance segmentation results of the test set, it can be seen that the LC-YOLOmatch with unlabeled data achieves an average improvement of 16.6% in the Mask(P) metric, 8% in the Mask(R) metric, 9.7% in the Mask (F1) metric, 14.2% in the *mAP*50 metric, and 9.8% in the *mAP*50-95 metric compared to the LC-YOLO model. Among them, the best performance is achieved when the ratio of labeled to unlabeled data is 1:2 and 1:3. An excessively high proportion of unlabeled data may introduce too much noise and lead to performance degradation. As shown in Figure 8, compared to the initial baseline model yolov11, our improved LC-YOLOmatch achieves a comprehensive performance improvement. We conducted tests using the publicly available standard laparoscopic cholecystectomy video provided by the Institute of Research and Innovation in Digestive Surgery (IRCAD) Cancer Center for the Digestive System in France (a globally renowned minimally invasive surgery training center) (Mutter and Marescaux, 2004). As shown in Figure 9, this figure presents the test scene from the surgical video.

**Table 9:** The impact of using LC-YOLOmatch on the results and the number of unlabeled images on the model.

**Class**	**label ratio**	**Images**	**Instances**	**Mask (P)**	**Mask (R)**	**Mask (F1)**	**mAP50**	**mAP50–95**
All	1:0	88	280	0.553	0.638	0.598	0.595	0.297
	1:2	88	280	0.647	**0.736**	**0.687**	0.698	0.395
	1:3	88	280	**0.683**	0.700	0.653	**0.700**	**0.408**
	1:8	88	280	0.655	0.631	0.629	0.640	0.382
Cautery	1:0	43	44	0.809	0.769	0.787	0.805	0.516
	1:2	43	44	0.787	0.756	0.771	0.819	0.550
	1:3	43	44	0.815	**0.841**	**0.828**	**0.856**	**0.597**
	1:8	43	44	**0.817**	0.773	0.795	0.831	0.576
Forceps	1:0	43	59	0.417	0.339	0.370	0.354	0.122
	1:2	43	59	0.615	**0.593**	**0.604**	0.523	0.255
	1:3	43	59	0.664	0.458	0.535	**0.556**	**0.282**
	1:8	43	59	**0.681**	0.362	0.461	0.462	0.214
Gallbladder	1:0	65	65	0.586	0.692	0.635	0.595	0.240
	1:2	65	65	0.600	**0.738**	0.663	0.712	0.333
	1:3	65	65	**0.676**	0.723	**0.699**	**0.781**	**0.390**
	1:8	65	65	0.565	0.646	0.603	0.541	0.274
Liver	1:0	78	108	0.559	0.731	0.638	0.654	0.345
	1:2	78	108	**0.653**	**0.824**	**0.730**	**0.754**	0.445
	1:3	78	108	0.532	0.778	0.634	0.681	0.423
	1:8	78	108	0.646	0.725	0.684	0.734	**0.470**

The optimal value is displayed in bold.

## 6 Discussion

The achievements made by the LC-YOLOmatch framework not only highlight the improvement in scene detection and segmentation capabilities during laparoscopic cholecystectomy, but also have significant implications for both theory and practice. From a theoretical perspective, our research results contribute to enhancing the multi-scale feature extraction and fusion capabilities of YOLO, providing new ideas for solving the theoretical problem of understanding complex surgical scene information, and opening up the integration method between YOLO and semi-supervised frameworks. At the practical application level, the optimization of this algorithm not only improves the accuracy of image recognition but also reduces the reliance on manual labeling. In the actual application of surgical assistance systems, it can effectively enhance the real-time performance and stability of the system, and has important application value.

During the semi-supervised experiment, we selected a certain number of unlabeled images each time, which might have had a certain accidental influence on the results. However, through multiple tests, we found that this algorithm could maintain a high accuracy rate in most cases, indicating that the performance improvement has a certain inevitability. From the perspective of the algorithm's principle, its precise extraction and efficient integration mechanism of features is a necessary factor for improving the recognition accuracy, which is consistent with our theoretical understanding of the image recognition process. When combined with our proposed semi-supervised framework, the model not only significantly improves the utilization efficiency of limited labeled data but also achieves better segmentation performance than these methods. It is important to note that this improvement relies on a large number of pseudo-labels and our specific semi-supervised strategy, and thus direct comparisons with fully supervised or models of different paradigms such as SAM2, SegFormer, and MedSAM are not strictly comparable or fair. Based on this, we have not included the above models in the main experimental comparisons for now. In future research, we plan to integrate these advanced models into our semi-supervised framework to ensure a more comprehensive and fair comparison under the same conditions.

**Figure 10:**
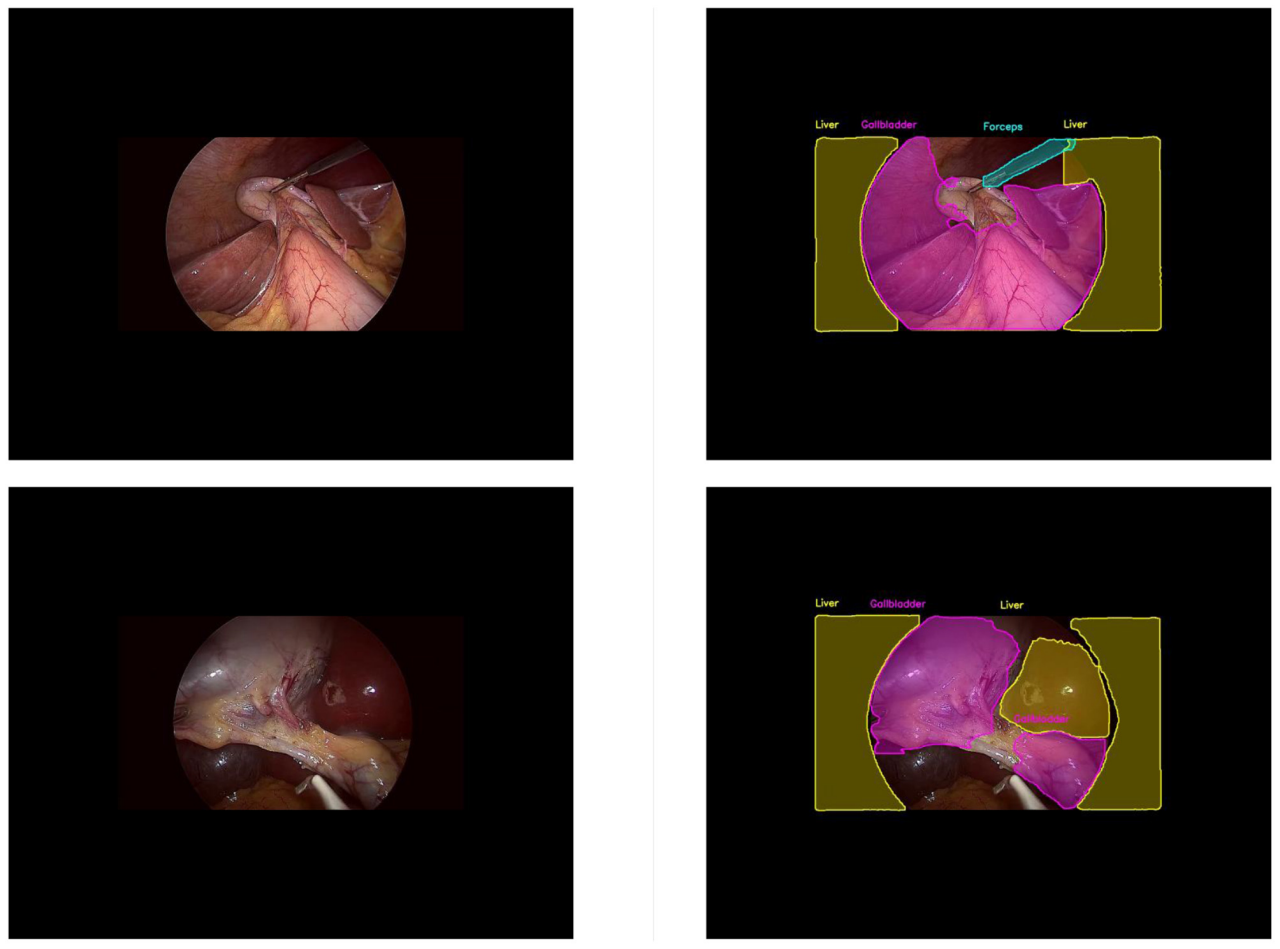
This is figure of common misjudgments in pseudo-label generation. **(Left)** unlabelled image data; **(right)** misjudged pseudo-label.

The experimental data set used in this study covers various categories, including major instruments and anatomical structures. In the actual surgical environment, the main clinical value of our model lies in its ability to continuously identify key anatomical structures, including the cystic duct, cystic artery, gallbladder boundary, and potential vascular injury areas, during the critical steps of establishing the critical view of safety (CVS). Although a 70% mAP50 might be considered moderate from a purely algorithmic perspective, our frame-by-frame error analysis indicates that the model provides stable detection results throughout most surgical stages, which is crucial for intraoperative assistance. In our test set, the optimized semi-supervised YOLO framework successfully identified the cystic duct area in over 85% of the visually exposed frames, while maintaining a low false positive rate of highlighting in regions without anatomical landmarks. This level of consistency can effectively support surgeons, especially those with less experience, by reducing the possibility of misidentifying the connection between the cystic duct and the common bile duct, thereby lowering the risk of major bile duct injury. Additionally, the semi-supervised enhancement method we adopted simulates the visual challenges similar to those in environments with heavy smoke, bleeding, or instrument occlusion, reflecting the actual surgical challenges and improving the model's stability. However, in real laparoscopic surgeries, there are more diverse instruments and anatomical structures, which may affect the generalization performance evaluation of the model in these scenarios. Since the experimental environment was set up for laparoscopic cholecystectomy, we were unable to fully test the algorithm's performance in other surgical systems, which may lead to certain deviations in our efficiency assessment of the algorithm in actual large-scale applications. Secondly, we used a unified threshold for filtering, which may not be suitable for all classifications. In multi-classification tasks, using a constant and uniform confidence level to filter pseudo-labels is difficult to balance the quantity and quality of labels to the optimal value. Furthermore, we found that as shown in Figure 10, our model often misclassifies the two arc-shaped quadrilaterals outside the laparoscope lens as liver. The possible reason is that the laparoscope lens is circular, while the captured image is rectangular. The receptive field around the lens is affected by the lens light source, and the gray value is not zero, so it cannot be identified as the background. In subsequent experiments, it may be necessary to preprocess the images to obtain better results.

Future research can combine this algorithm with other more advanced semi-supervised frameworks to further enhance its adaptability and intelligence level. Additionally, the application of this algorithm in more complex surgeries, such as laparoscopic gastric cancer radical surgery, can be explored to provide further technical support for surgical automation.

## 7 Conclusions

In summary, LC-YOLO-match has made significant progress in the automatic segmentation task of laparoscopic cholecystectomy scenarios. By introducing the BiFPN module and the DWRseg module, LC-YOLO not only achieved breakthroughs in high detection rate and segmentation accuracy, outperforming multiple baseline models, but also maintained a low number of parameters and GFLOPs. Additionally, we proposed the LC-YOLOmatch framework, which effectively utilizes unlabeled data to alleviate the problem of scarce labeled data. Experimental results show that this method achieved 70% *mAP*50 and 40.8% *mAP*50-95, surpassing the previous best levels of 55.6 and 28.4% respectively, reaching a new technical level. These results highlight the potential of customized deep learning methods in automated surgery, especially in achieving high performance with limited manually labeled data. The outstanding performance of LC-YOLO-match not only provides a direction for future improvements but also lays the foundation for further integration into clinical workflows, ultimately contributing to assisting surgeries and reducing the occurrence of complications.

## Data Availability

The datasets presented in this study can be found in online repositories. The names of the repository/repositories and accession number(s) can be found in the article/supplementary material.
